# The family meal, a ritual frozen in time; an Australian grounded theory study

**DOI:** 10.1093/heapro/daad124

**Published:** 2023-10-04

**Authors:** Georgia Middleton, Rebecca K Golley, Karen A Patterson, John Coveney

**Affiliations:** Caring Futures Institute, College of Nursing and Health Sciences, Flinders University, Tarntanya, Adelaide, South Australia, Australia; College of Nursing and Health Sciences, Flinders University, Tarntanya, Adelaide, South Australia, Australia; Caring Futures Institute, College of Nursing and Health Sciences, Flinders University, Tarntanya, Adelaide, South Australia, Australia; College of Nursing and Health Sciences, Flinders University, Tarntanya, Adelaide, South Australia, Australia; College of Nursing and Health Sciences, Flinders University, Tarntanya, Adelaide, South Australia, Australia; Caring Futures Institute, College of Nursing and Health Sciences, Flinders University, Tarntanya, Adelaide, South Australia, Australia; College of Nursing and Health Sciences, Flinders University, Tarntanya, Adelaide, South Australia, Australia

**Keywords:** family meal, grounded theory, food work, food provision, time comparison

## Abstract

Family meals are recognized as an opportunity to promote the health of families. Popular discourse posits that changes to contemporary family life have made family meals harder to achieve and promotion of the ‘traditional’ family meal may be adding pressures to contemporary families. While research has been conducted on family meals over the last three decades, there is no explicit investigation of the experiences and practices of family meals over this time. Understanding the evolution of family meal practices across time is important for developing achievable expectations in relation to this ritual. Qualitative interviews were conducted with a diverse population of South Australian parents in the 1990s (*n* = 32) and with a separate population of parents in 2020 (*n* = 22) to gather their experiences of family meal practices. A comparative analysis, informed by grounded theory, was undertaken to identify similarities and differences in experiences across these two time periods. The results indicated stability in many family meal experiences across time, particularly in their value and significance in family life. Negotiations balancing time, cost, food preferences and responsibility persisted. The stability of family meal values and practices is important to consider when making recommendations, designing interventions and creating services targeting the family meal.

**Contribution to Health Promotion**

- Family meals are an important area of focus for health promotion, as an opportunity to improve the health of families and children globally.
- Understanding how family meals have changed over time helps us to promote them more appropriately and realistically.
- This work aims to support health promotion through recommendations to change provision of supports and resources to families, and social policy changes to ensure access and opportunity for parents and families to come together for a shared meal.

## INTRODUCTION

The ritual of sharing food together, or ‘commensality’, has deep cultural roots and proposed benefits for social, mental and physical health (Ochs & Shohet, 2006; Dunbar, 2017; Lindsay et al., 2019; Jönsson et al., 2021). Family meals are shared eating occasions that can provide opportunities for role-modelling and social learning around eating habits and behaviours, and manners in children (Martinasek et al., 2010; Quick et al., 2011; Berge et al., 2013; Momin et al., 2014; Trofholz, Schulte, et al., 2018). They provide a recurring setting with consequences for diet quality and development through childhood into adolescence (Fiese & Schwartz, 2008). Additionally, the family meal has been recognized as an integral part of family life (Murcott, 1995; Charles & Kerr, 1988; DeVault, 1991; Walton et al., 2020), and a means of keeping family relationships connected. It is for these reasons that the family meal has been consistently researched over the last few decades, and widely promoted through public health and media messaging as an activity families should regularly engage in (Le Moal et al., 2021).

The common narrative around family meals today posits that it is becoming more challenging for families to maintain this tradition (Bowen et al., 2019). The last three decades have seen significant shifts in working and family life. More women are working outside of the home, with trends showing a 27% increase in Australian mothers entering the paid workforce since 1981, commonly in part-time positions, with similar rates seen across the Western world (Australian Bureau of Statistics (ABS), 2017; Australian Institute of Family Studies (AIFS), 2010). The number of stay-at-home mothers has decreased (Australian Institute of Family Studies (AIFS), 2018). The number of dual-employed families has increased (Australian Bureau of Statistics (ABS), 2017). With these changes to family life, family routines have changed and, in many instances, have resulted in increased time pressures and conflicting schedules (Fulkerson et al., 2011; Berge et al., 2013; Brannen et al., 2013; Bacon, 2018; Skeer et al., 2018).

These changes to family life have been proposed as resulting in changes to family meal frequency and environment. Families have reported relying on convenience foods, eating on the run, in front of the television or at separate times (Quarmby & Dagkas, 2013; Alm & Olsen, 2017; Litterbach et al., 2017; Trofholz, Thao, et al., 2018). These constructions are at odds with the ‘ideal’ family meal promoted across Western society (Le Moal et al., 2021). This is problematic as an inability to live up to the expectations of the ideal ‘traditional’ family meal—where parents and children gather together in the same place, at the same time, to consume the same meal that has been prepared in the home from whole ingredients (Charles & Kerr, 1988; DeVault, 1991; Daragan et al., 2023)—can lead parents to feelings of shame and guilt around parenting and food provision practices (Kling et al., 2009; Kinser, 2016). Furthermore, the family meal is positioned as a time to impart knowledge, values and social cues, provide food for adequate growth and development and to spend time together as a family (Martinasek et al., 2010; Quick et al., 2011; Berge et al., 2013; Malhotra et al., 2013; Quarmby & Dagkas, 2013; Skeer et al., 2018). The additional burden of family meals serving all these purposes at once may in fact be causing more harm than good.

This pressure may be further perpetuated by the comparison of family meal practices now, against family meal practices of the past. Contemporary discourse posits that changes in society have resulted in a shift away from the ‘traditional’ family meal at the expense of family and children’s health (Wilk, 2010). However, we do not have evidence that this is explicitly the case. Authors have argued that the family meal has long been asserted as an ideal, and not a certifiable institution found in every home in the past (Murcott, 1997), and that the ritual of coming together for the family meal has been more variable over history than acknowledged by those proclaiming its decline (Murcott, 1997; Jackson et al., 2009; Oleschuk, 2020). However, contemporary families are still judged, and judging themselves, against these depictions of a family meal ideal that likely never existed.

In summary, we know that family meals are promoted as a positive activity for families. We also know that many families struggle to achieve the idealized version of the family meal. However, what we do not know, is how changes to society have impacted families’ constructions and experiences of family meals over time. While there have been explorations of family meal experiences over time (Jackson et al., 2009; Brannen et al., 2013; Trofholz, Thao, et al., 2018; Hammons et al., 2020), this research is dominated by time-diary comparisons, archival analyses, short-time periods of investigation and retrospective reflections. This study aimed to fill this gap by exploring how family meals have changed over the last 30 years, to better position family meal recommendations.

## STUDY DESIGN

A comparative qualitative study, informed by grounded theory was undertaken. The consolidated criteria for reporting qualitative research (COREQ) 32-item checklist for interviews and focus groups were used to prepare this manuscript (Tong et al., 2007).

## METHODS

### Philosophical position and methodology

This study was informed by social constructionism; the position that meaning is constructed by humans through interacting with the world around them (Burr, 2015). From this position, we acknowledge that meaning and knowledge are subjective and value-laden (Crotty, 1998; Burr, 2015). The results presented in this article are part of a larger grounded theory study that aimed to construct a theory around the experiences, processes and considerations of family meals over the last 30 years.

The terms ‘family’ and ‘meal’ are not defined by a set of fixed criteria. For the purposes of this study, ‘family’ included immediate family members consisting of parents and children, biological or otherwise, and ‘meal’ included any intentional eating occasion with substantial food consumption.

### Data source

This study was intentionally designed to be a comparative study of family meal practices across two distinct time periods. The data sources for this analysis were the qualitative interview data from South Australian (SA) parents in the 1990s and 2020. More detailed methods for each dataset are published elsewhere (Coveney, 2004; Middleton et al., 2022) but are described briefly.

In 1993–94, qualitative interviews were conducted with parents from two diverse SA suburbs by J.C., a doctoral candidate, dietitian and lecturer at the time (referred to as the ‘1990s’ sample). Families comprised of no more than four children, with at least one child aged ≤ 12 years of age. Interviews were conducted using a semi-structured guide, in participant’s homes, audio recorded and transcribed verbatim. They explored family food practices, including the family meal, and three interviews were conducted with each family, two with both adults present.

Families from the 1990s dataset (*n* = 40) were purposively sampled, following theoretical sampling methods (Charmaz, 2014), to ensure a diverse representation of different family types and to reach theoretical saturation. Families were selected according to their family composition (e.g. single- or dual-parent household, number and ages of children, employment status of parents) and their transcripts were analysed prior to the selection of a new family from the sample (Charmaz, 2014). This continued until theoretical saturation was achieved (Charmaz, 2014). All 1990s data were analysed prior to conducting the 2020 interviews and there was no connection between 1990s and 2020 participants.

In 2020, interviews were conducted with parents from two diverse SA areas by G.M., a doctoral candidate and dietitian. Eligibility criteria mirrored the 1990s sample. Interviews were conducted using an adjusted version of the 1990s semi-structured guide, virtually via Zoom, audio recorded and transcribed verbatim. They focussed on family meal practices; one interview was conducted with each family with both adults present.

In 2020, families were recruited into the study based on the same criteria of diverse family compositions. Theoretical sampling was conducted until theoretical saturation was achieved.

### Data analysis

Interview transcripts were analysed according to grounded theory methods (Charmaz, 2014). This included line-by-line coding, focussed coding, development and refinement of categories and development of the theory (Charmaz, 2014). G.M. conducted an analysis and drew on the combined experience and expertise of the research team. Datasets were analysed separately and resulted in the development of the Family Meal Framework (Middleton, et al., 2022). The framework provided the categories for comparative analysis; the codes that sat within the categories, and the data that sat within the codes, were compared to determine similarities and differences between the two time periods.

For effective comparative research and re-use of existing datasets, the comparison must be a defining component of the study design, units of comparison must be clearly delineated, data functionally equivalent, the context of original and current use of data provided and reflexivity practised (Jackson et al., 2008; Esser & Vliegenthart, 2017; Phoenix et al., 2021). For this analysis, comparison is the defining component of the research, the units of comparison (interview transcripts) are clearly delineated and the data functionally equivalent (data within the framework). Contextualization of both datasets has been provided by describing data-collection methods and providing background context (Supplementary file S1). Both datasets were analysed separately and contextualized within their own time period. Reflexive practice, in accordance with grounded theory, was practised across all stages of the project. J.C., who collected the 1990s data, was heavily involved in this project.

## RESULTS

The main differences and similarities in the construction and practices of family meals as experienced by parents across time are presented thus: stability in the practices and value of the family meal over the two time periods; managing considerations of time, money and family food preferences across time; evolution of responsibility for the family meal over time.

### Participants

A total of 32 adults from 16 families in the 1990s, and 22 adults from 12 families in 2020 participated. As per our sampling methods, we achieved diversity in our two samples. Of the 32 participants in the 1990s sample, 16 identified as male, and 10 of the 22 participants identified as male in the 2020 sample. The mean age of participants was 38 years in the 1990s sample (range 26–46 years) and 43 years in the 2020 sample (range 34–55 years). There was a higher proportion of participants who had secondary school as their highest level of education in the 1990s (13/32) compared to the 2020 sample (2/22). The majority of 2020 participants held a degree or tertiary diploma (16/22), which was the second-highest category of education for participants in the 1990s sample (10/32). Most families consisted of two parents who were either married or in a domestic partnership, with one single-parent household in the 1990s sample and two in the 2020 sample. A greater proportion of participants, both men and women, were employed in the 2020 sample (20/32 in the 1990s, 19/22 in 2020), and consequently, there were more households in the 2020 sample where two parents were employed (7/16 families in the 1990s, 8/12 families in 2020). In the 2020 sample, fewer participants self-identified as ‘homemakers’ (7/32 in the 1990s sample, 2/22 in the 2020 sample) or self-identified as ‘unemployed’ (5/32 in the 1990s, 1/22 in 2020). The average number of children per household remained consistent across the two samples at 2.4 (range 1–3 in the 1990s, 1–4 in 2020). Children’s ages ranged between < 2 and  > 18 years, with children between the ages of 3 and 15 making up most household compositions in both samples (>80%). Participants’ annual household income ranged from the highest quintile to the second quintile across both samples. Detailed participant demographics are presented in Supplementary file S2. Participants have been given pseudonyms and family identification codes (e.g. F1 = Family 1).

### Stability in the practices and value of the family meal over the two time periods

Components of what constituted a ‘family meal’ remained consistent over the two time periods. The identification of components participants considered integral to the family meal, was best captured when discussing the eating occasions they did not consider to be a ‘family meal’. Lack of substance, formality, intention or communication distinguished other eating occasions from the ‘family meal’. Therefore, the defining components of a ‘family meal’ were the presence of most family members in the same location with the intention of eating a meal and spending time together. Across both time points, this would typically occur once a day most commonly in the evening.

1990s:

Colin: Everybody tends to be together in the one place at the one time and people tend to be a bit more relaxed, so it tends to be the meal which we congregate. (F12, father employed, stay-at-home mother, two children aged 9 and 7 years old)

2020:

Griffith: It’s not formal, but it’s more… it’s an opportunity for everyone to be together, again for a more hearty meal, and the experience, the connection with food and people. (F26, both parents employed, two children aged 9 and 4 years old)

Family meals occurred in the living room or kitchen/dining room over the two time periods. Technology use was mixed with several families across the two time points consistently eating their family meals in front of the television, others doing so occasionally, and some adamantly avoiding it.

1990s:

Mary: No most of the times we eat in the lounge and watch TV. (F3, father unemployed, stay-at-home mother, three children aged 15, 11 and 8 years old)

Frances: I would never want to be a family that ate while they watched TV. (F12, father employed, stay-at-home mother, two children aged 9 and 7 years old)

2020:

Scott: We’re all sort of sitting next to each other, just in the loungeroom.

April: Yeah, around the TV. (F18, both parents employed, two children aged 5 and 2 years old)

William: There’s not normally any TV or anything on… we just sit at the dinner table, and yeah there’s normally no distractions, or TV, or phones, or anything. (F21, both parents employed, three children aged 15, 13 and 11 years old)

There was no clear indication of technology use increasing or decreasing across the two time periods. Some participants described technology as having a negative impact on the family meal environment or their ability to communicate with one another. Others enjoyed the entertainment or distraction it brought. Regular technology users negotiated ways it could positively enhance their eating experience.

1990s:

Tim: We’re going to build a cabinet and just have it away, the TV… we can sit around and talk, and we don’t. It’s beginning to be a problem with the television there. (F10, father employed, stay-at-home mother, two children aged 3 and 1 years old)

2020:

Helena: It [watching a show] helps the youngest child… he’s a kinetic person, so he can do lots of things at once, and he likes movement and everything, so that can keep him at the table, and engaged with us. (F24, single-mother family, stay-at-home mother, three children aged 24, 12 and 10 years old)

Family mealtimes were not always easy to manage and did not always align with expectations across both time points. For participants with young children, disruptive behaviours, such as food refusal, fighting with siblings or attempts to leave the meal, were frequently encountered. As children got older, their increasing independence impacted their ability to share meals together.

1990s:

Brooke: While they’re sitting there eating they won’t, they’re thinking about something else and go and off and do it, and it’s quite a job just to keep them sitting down on the seat and eating at this age. (F10, father employed, stay-at-home mother, two children aged 3 and 1 years old)

Vera: We’re not all the time here because every, sometimes like Filip [son] is waiting you know, stayed longer or something, we’re not waiting for him, we have meal without him, so he’s missing and we’re missing you know on this togetherness. (F8, both parents employed, three children aged 19, 15 and 10 years old)

2020:

Christopher: We do occasionally have to, like, put our foot down though and say, ‘no you have to try that, you have to try this’. You know, ‘cause it does get ridiculous sometimes. (F22, both parents employed, two children aged 6 and 4 years old)

Donna: As the kids are getting older now, their own independence, so, they’re definitely the barriers to us having more frequent dinners. (F27, both parents employed, three children aged 20, 18 and 8 years old)

The value parents ascribed to family meals was consistent across the two time periods. In most instances, participants saw them as valuable and important family time, an opportunity not to just eat a meal, but to communicate, connect and check-in with one another. Participants could not identify another time or activity that could serve these purposes simultaneously, or as effectively.

1990s:

Mara: Talk about you know, what we’re going to do on the weekend, how things are going to fit in, or what they’ve done at school, those sorts of things. (F13, both parents employed, three children aged 13, 12 and 10 years old)

2020:

Melanie: So, that’s what’s so special about it, because it’s this one united time where you can sit down, reflect on each person, how they’re going too, like almost like a check-in, see how the weeks’ going. It’s a really unique time that, and there’s no distractions, it’s special in that sense. I think that that’s what makes it different, because we’re all there, all doing the same thing, all engaged. (F28, both parents employed, two children aged 11 and 7 years old)

Not all participants saw this value and were satisfied eating separately from one another. There was more discussion of parents eating separately from their children out of preference in the 1990s sample. This was evident when family dynamics produced unpleasant environments, or when they did not result in feelings of connection as expected.

1990s:

Rose: Vic [partner] doesn’t like eating with children because it’s reasonably stressful, we joke that it gives him indigestion, that’s a joke, so that’s basically why we don’t. (F16, father employed, stay-at-home mother, two children aged 6 and 3 years old)

2020:

Julianne: The things that I see as more important than having that group time, is having one-on-one time with the kids… having one-on-one moments with the kids is more important to me than having that [the family meal]. (F20, father employed, stay-at-home mother, four children, one aged 7 and triplets aged 6 years old)

### Managing considerations of time, money and family food preferences across time

The time parents had available for family meal tasks, such as shopping and cooking, was a major consideration for most participants across the two time periods. Most participants in the 1990s, and almost all participants in 2020, factored time into their family meal decisions. Even if time was not a limited resource, no participant wanted to spend more time than necessary preparing a family meal.

1990s:

Angela: The rest of the week when I’m picking them up from school I usually get back with them about 4 o’clock and that kind of cuts down the amount- we would rather have dinner by five…So it cuts down the amount of cooking time. (F1, both parents employed, three children aged 15, 8 and 5 years old)

2020:

Jennifer: There is definitely, I wouldn’t call it time pressure, but we’re just aware that it needs to happen in the next hour and be over and done with and we need to get out the door. (F17, father employed, mother volunteers, four children aged 19, 18, 13 and 11 years old)

Across the two time periods, the main strategies participants discussed to help manage time were preparing meals in bulk or in advance, using leftovers, purchasing takeaway, using convenience foods or outsourcing cooking to grandparents. The use of these strategies varied across the two time periods, with most only being used sporadically by one or two families. However, there was more discussion from participants in 2020 about preparing meals in advance and serving takeaway meals, and more discussion in the 1990s about using convenience food items, particularly frozen meals.

1990s:

Mara: The little pasta place… and they will sell you their frozen stuff at a lot cheaper than their fresh… And they might have a couple of lasagnes, they might have some ravioli or whatever. (F13, both parents employed, three children aged 13, 12 and 10 years old)

2020:

George: If I’m home sometime during the day, you try to get as much done as you can so that when you get home from school and you’ve got a ballet lesson, or a guitar lesson and soccer, you’ve got all those things, it’s not really a good time for, between say three-thirty and six, it’s not a great time for organising things. (F19, both parents employed, two children aged 8 and 5 years old)

Richard: Let’s say we can’t be stuffed and you know it’s been a difficult day, we might just go and get Domino’s or something. (F17, father employed, mother volunteers, four children aged 19, 18, 13 and 11 years old)

While new services and technology were available in 2020, such as online grocery shopping and meal box schemes, uptake of these was minimal. Only three participants in 2020 routinely used online grocery shopping, and only one used a meal box scheme regularly. The cost was a deterrent for using meal box schemes for most parents, and others did not perceive the meals as suitable for children.

Claire: Once in a while, we have subscribed to like (mealbox scheme) and I’ll get the veggie box ones, generally just when they have good special on them… And then that’ll completely change things up, ‘cause then Chris [partner] and I will eat whatever that meal is and I’ll do something separate for the kids. But that’s not our norm, that’s just once in a while we’ll do that. (F22, both parents employed, two children aged 6 and 4 years old)

Julianne: I’ve always kind of done online shopping. (F20, father employed, stay-at-home mother, four children, one aged 7 and triplets aged 6 years old)

Within each time point, cost appeared to be a priority for most families. Some participants in the 1990s experienced limitations on the quantity, quality and variety of foods because of the high cost. In 2020, almost all participants considered the cost of food; however, none described feeling limited in their choices in the same way.

1990s:

Audrey: No, I don’t think that if I had more money to go and shop for food they’d be eating like kings every day. I would go out and buy more fish and chicken and porks-

Dean: Roasts.

Audrey: -roasts and all that sort of stuff. No, I think we’d just eat a lot better than what we’re eating now. (F15, both parents employed, one child aged 10 years old)

2020:

George: Food has become expensive. I don’t know when it happened, but you sort of notice over time, you used to fill a basket up for twenty or thirty dollars, and now you can get up to eighty dollars just on a basket of food. And I don’t know it might be the quality or trying to by organic, or fresh, or local produce, that does that. (F19, both parents employed, two children aged 8 and 5 years old)

Consequently, compared with the 2020 families, several participants in the 1990s spoke about engaging in budgeting practices to keep within spending limits and chasing bargains to keep costs down.

1990s:

Meg: I often, because of my husband’s wages not too much [sic]… I have to thinking [sic] something depend on I need for every week… I separate the money and just about fifty for the children’s breakfast… and meat and something like that about one hundred dollars. (F9, father employed and studying, stay-at-home mother, three children aged 11, 9 and 3 years old)

2020:

Helena: To source out cheaper deals… at the moment we’re ok and I, financially I don’t have to. (F24, single-mother family, stay-at-home mother, three children aged 24, 12 and 10 years old)

The practice of planning meals or writing shopping lists in advance was not commonly employed across either time point. Very few participants engaged in any structured planning processes, however, there was more discussion about planning meals in 2020.

1990s:

Martin: Sometimes Maureen [wife] plans what we should be having probably a day in advance maybe… ‘cause food doesn’t last very long in the fridge so you never know what’s going to happen… but generally it’s just work it out on the day. (F7, both parents employed, three children aged 8, 7 and 4 years old)

2020:

Evana: Going through my recipes and seeing what ones I will do, and what the kids will actually eat, and doing the shopping for it and then yeah, then designating which night to cook it. (F26, both parents employed, two children aged 9 and 4 years old)

Another key consideration across the two time periods was children’s food preferences. While not all participants entirely accommodated their children’s preferences, most adjusted what was prepared to appease them. These considerations were often juxtaposed by desires to expand children’s palates, and fuelled by fears of disrupted growth and development if children did not eat enough food.

1990s:

Alison: I think our biggest concern is that they don’t eat really, seem to eat enough, in the case of Bonnie [daughter]. (F4, father unemployed, mother employed, two children aged 9 and 7 years old)

2020:

George: So there’s some [foods] they [children] definitely won’t eat, and then I s’pose there’s the ones that they’d prefer not to and will, yeah, will try and push that aside to get something else… There’s definitely different tastes and yeah, that needs to be sort of catered for. (F19, both parents employed, two children aged 8 and 5 years old)

To accommodate children’s food preferences across both time periods participants prepared separate meals. However, this strategy was more consistently employed by participants in the 1990s, so that both children and adults could eat a meal they enjoyed. Contrasting this were the 2020 participants who were either more willing to compromise their own preferences for the sake of only having to prepare one meal or were more insistent that their children eat what was served.

1990s:

Brooke: I’m finding it’s easier for me to provide them [children] with a separate meal and then have my own meal, than it is to provide a meal that we’ll all eat, because they eat a blander meal… and Tim [partner] and I want some variety in our meals. (F10, father employed, stay-at-home mother, two children aged 3 and 1 years old)

2020:

April: I guess for us, we’re pretty flexible with what we want to eat, so if it means cooking one meal over two meals then we’ll sort of go with, go with that. (F18, both parents employed, two children aged 5 and 2 years old)

### Evolution of responsibility for the family meal over time

The expectations and division of responsibility for family meals present the biggest evolution. It was expected by participants in the 1990s that women would take responsibility due to working fewer hours outside of the home, and as men typically worked full-time, they were precluded from these tasks. When men in the 1990s did take on responsibility, they were lauded for going against these expectations. This sentiment was not expressed by 2020 participants.

1990s:

Martin: I have to work 15 or 16 hours a day so I can’t be expected to do the shopping, cleaning, do anything like that, I wash the dishes occasionally, more than often, on the weekends I wash them, sometimes in the morning’s I clean up and all this sort of thing, so I do my bit. (F7, both parents employed, three children aged 8, 7 and 4 years old)

Sylvia: They [other women] just think I’m lucky, I don’t know. You know, not many of them that I know that the husbands will do a lot of the work in the house, so I’d say we’re a bit different. (F5, father employed, stay-at-home mother, two children aged 6 and 3 years old)

Aligned with the shifts in expectations, there was also a shift in the descriptions of the division of responsibility for family meals across time. Families in the 1990s predominantly had one parent, the woman in every case, responsible for family meals. In most cases, the men were either not involved or held a ‘supporting’ role. In 2020, it was common in two-parent households for both parents to share responsibility, and the alternative ‘supporting’ role was not evident.

1990s:

Harry: If Mara’s [partner] busy, then I might sort of half prepare a meal. Like Mara might prepare it and she’ll say, ‘just cut up the veggies and put them in the wok or microwave them’, or whatever, and then I’d sort of put everything in the oven… I’d be doing something towards finishing the meal off and rarely I’d make a complete meal. (F13, both parents employed, three children aged 13, 12 and 10 years old)

2020:

Interviewer: Who normally cooks the family meal in your household?

Leslie: It’s both of us depending on who’s available.

Joaquin: Who has time yeah. (F25, both parents employed, one child aged 12 years old)

Additionally, the reluctance of women to allocate family meal tasks to their partners remained persistent. Across both time points, men expressed a desire to be involved, however, they were met with resistance from their partners. This resistance was due to the perceived incompetence of the men to perform the tasks adequately or to women’s standards. While 2020 saw fewer instances of women not trusting their partners to undertake these tasks, these sentiments pervaded for some.

1990s:

Gaye: He [partner] would go to the delicatessen part and he would come back with several different items like salami and liverwurst and ham and things like that and he’d have about three or four of them and he’d bring them home. And I’d say, ‘how are we going to get through them in a week?’. (F2, both parents employed, two children aged 11 and 8 years old)

2020:

Claire: It’s pretty much always me [that does the shopping].

Christopher: Yeah, pretty much.

Claire: Not ‘cause he doesn’t want to help, but because I don’t like him helping with that. (F22, both parents employed, two children aged 6 and 4 years old)

## DISCUSSION

The findings from this comparison of parents’ perceptions of family meals in the 1990s and 2020 indicate the stability of the construction and value of family meals, the evolving trade-offs of time, money and food preferences, and the evolution of who bears responsibility for their execution. To the authors’ knowledge, this is the first study that recreated interviews to generate a comparable data set of family meal practices across two time periods and to do so in an Australian context.

There was stability across the two time periods regarding dedication to regularly bringing the family together for a meal. The way family meals were executed also remained stable with families engaging in similar practices and processes across the two time points, even as time and scheduling pressures increased. This rhetoric of the importance and value of the family meal, regardless of its achievability, was evident across both time points. Participants acknowledged the role of family meals in their ability to bond as a family and feared for their connection as a family unit without them, consistent with recent literature (Wasche et al., 2021). While this notion of positive connection at the family meal is not implicitly negative, the inference behind these assumptions, and as described by our participants, is that it is one of the only ways to ‘enact’ family (Wilk, 2010; Meah & Jackson, 2013). There were families across both time points who did not view the family meal as a positive experience, experiencing disruptions by children and feeling disheartened when their older children were no longer able to participate. Thus, the idealization of the family meal as an inherently positive experience, pervasive across time, has likely created unrealistic expectations of how and why the family meal should be executed and may add unnecessary pressure on parents (Wilk, 2010; Meah & Jackson, 2013).

Additionally, the family meal is promoted as a time for parents to impart knowledge, values, social cues and manners to their children, and an opportunity to provide them with the nourishment needed to develop and grow (Martinasek et al., 2010; Quick et al., 2011; Berge et al., 2013; Malhotra et al., 2013; Quarmby & Dagkas, 2013; Skeer et al., 2018). The associations between family meals and health outcomes place additional pressures on parents to ensure not only that family meals occur regularly but that they are healthful, positive and instructional. Family meals are moralized as the ‘right thing to do’ for children’s health, but the burden is consistently placed on parents to achieve them (Oleschuk, 2020). This was evidenced by participant’s trade-offs between desires to expand their children’s palates by exposing them to new foods, against fears of their children’s growth and development suffering if they did not eat their meals, as seen in prior literature (O’Connell & Brannen, 2016a). Participants across time employed different strategies to deal with these competing priorities. For example, 1990s parents discussed preparing separate meals to cater to different preferences. In 2020, parents discussed preparing one meal that aligned with their own or their children’s preferences over preparing separate meals, likely as a result of the increasing feelings of time pressure experienced by contemporary parents (Strazdins et al., 2016; Alm & Olsen, 2017). These trade-offs add to the cognitive load of parents and further exacerbate the gap between expectation and practice.

The gap between family meals as an ideologic ritual and as a realistic activity was evidenced by the enduring pressures of time, money and family members’ food preferences across the two time points. Our findings demonstrated that all three of these factors were commonly and consistently considered across time, albeit at varying levels of priority, often requiring trade-offs to make the family meal achievable. While these challenges are acknowledged in media and research, the strategies that are promoted to overcome them, such as engaging in meal planning, using shopping lists, purchasing and cooking foods in bulk or utilizing leftovers (Fiese et al., 2012; Berge et al., 2013; Alm & Olsen, 2017; Loth et al., 2019), were rarely used consistently or effectively by participants across either time point. Contemporary solutions are offered in online services and technology (Hertz & Halkier, 2017; Fuentes & Samsioe, 2020; Laila et al., 2023), and prior research has indicated that these services can reduce time burden and simplify meal preparation (Hertz & Halkier, 2017; Fuentes & Samsioe, 2020; Oberle et al., 2020; Carman et al., 2021; Fraser et al., 2021; Laila et al., 2023). However, the use of such services and technology was scarce among our contemporary sample. These strategies are quick-fix solutions to the systemic problems of time scarcity, financial instability and conflicting food preferences, and their inconsistency indicates that they are not adequate solutions.

The family meal cannot be discussed without acknowledging the burden of responsibility placed on women to undertake it. This presents the largest disparity between the two time periods. The 1990s data indicated women as primarily responsible for family meal tasks, and the 2020 data indicated that more men were involved and taking some degree of responsibility for these tasks. However, the gendered divide of responsibility remained in some 2020 households regardless of dual-employment status, aligning with recent literature (Burnod et al., 2022; Storz et al., 2022). Furthermore, as we did not observe these practices, we cannot definitively state that men’s participation has increased, but that the discourse between couples and the presentation of family meal practices may have changed. Women, traditionally undertaking food work, have been ascribed with gender traits of nurturers and carers, and thus these behaviours and expectations become inextricably linked (Eagly & Wood, 2016). This has created tensions for mothers when they choose to or must enter the paid workforce, whereby they must contend with the social expectation of providing their full attention to their family, whilst concurrently performing at the highest standard at their workplace (Offer, 2014). Being a ‘good’ mother is fraught with moral responsibility and expectation that is not prevalent for fathers (Offer, 2014; Epp & Price, 2018; Mehta et al., 2019). While the notions of ‘good fathering’ are shifting, there is not the same level of moral responsibility, and this emotional burden appears to be prevalent for mothers alone (Offer, 2014; Epp & Price, 2018; Mehta et al., 2019). This study also confirms previous findings regarding women’s reluctance to hand over the responsibility of food work tasks to men due to perceived incompetence, or reluctance to hand over control (Cinotto, 2006; Beagan et al., 2008; Curtis et al., 2009; O’Connell & Brannen, 2016b; Burnod et al., 2022). Without structural and societal shifts regarding the expectations of men’s and women’s roles and responsibilities at work and home, women will likely continue to bear the burden of this work.

### Strengths and considerations

This study is strengthened by its use of contextualized historical data to compare family meal experiences across time. The units of the comparison were comparable by design and the use of grounded theory methods strengthened the rigour and reliability of this work (Charmaz, 2014). This research also included a close to equal proportion of men in the sample, an underrepresented population in family meal research (Metcalfe et al., 2009; Khandpur et al., 2016) and explores experiences of family meals in households of varying compositions.

The main considerations to situate this analysis are the potential risks of self-selection and social desirability bias of participants in both samples. A specific limitation of this comparative analysis was that some topics were explored in more depth with one sample than the other. Furthermore, this study explored family meal experiences at two separate time points but did not investigate family meals between these two times and did not incorporate children’s perspectives.

## CONCLUSION

Family meals in general, where, and how they took place, and the value placed on them have not changed considerably over the last three decades, regardless of the increasing pressures placed on contemporary families. The contemporary solutions we do have are not working to adequately support parents against these pressures. If we are to continue using the family meal as a vehicle for promoting health, nutrition and family connection, we need to construct systems that support families to achieve realistic versions of the family meal that are achievable. This may include providing services and technology that meet parent’s needs, such as flexible workplace policies, nutritious and affordable family-friendly convenience meals, or school or workplace programs that provide meals to take home and share with the family. Importantly, we need to shift the way we construct family meals and the societal and moral burden we place on women to hold responsibility for feeding the family.

## Supplementary Material

daad124_suppl_Supplementary_Files_1

daad124_suppl_Supplementary_Files_2

## Data Availability

The data are not able to be made openly available to the public due to the nature of the data, the conditions of ethics approval, and privacy concerns. Data may be made available via a request to the authors in which a formal data-sharing agreement may be required.
