# Supplementary material for: The family meal, a ritual frozen in time; an Australian grounded theory study
Source: Health Promot Int. 2023 Oct 4;38(5):daad124. doi: 10.1093/heapro/daad124 (PMC10548410; doi:10.1093/heapro/daad124)
Supplement: daad124_suppl_Supplementary_Files_1 [file daad124_suppl_supplementary_files_1.docx]

Supplementary File 1:

# Background context to situate the comparative analysis

Over the last three decades working life, family life, technology and services have changed for Australian families. The median age of adults in Australia increased from 32 to 41 years, and the median age of adults in SA increased from 32 to 45 years (Australian Bureau of Statistics (ABS), 2010, 2019; Castles, 1993). Median annual income has increased over this time by ~AU$25,000 nationally, and by ~AU$28,500 for families in SA (Australian Bureau of Statistics (ABS), 2016, 2017a; Castles, 1993; Greenville et al., 2013). The number of children per household has remained relatively consistent at 1.9 in 1992 and 1.8 in 2016 (Australian Bureau of Statistics (ABS), 1995, 2016, 2017a). Rates of single-parent households has remained stable at ~14% for Australian families between 1992 and 2016 (Australian Bureau of Statistics (ABS), 1995, 2020). However, single-parent households increased by 4% for SA families over this same time, representing 16.5% of households in SA by 2016 (Australian Bureau of Statistics (ABS), 2016; Castles, 1993).

Perhaps one of the most relevant changes to situate the results of this comparative analysis are the changes to work and household arrangements between the 1990s and 2020. In SA, rates of women entering or re-entering the paid workforce continued to rise from 50% in 1990 to 54.9% in 2020, and rates of stay-at-home mothers decreased from 32% in 1991 to 24% by 2016 (Australian Bureau of Statistics (ABS), 1994; Castles, 1993; Warren et al., 2020). Consequently, the rates of households with dual-employment increased by 20%, representing 68% of two-parent households by 2016 (Australian Bureau of Statistics (ABS), 1994; Castles, 1993; Warren et al., 2020). However, while women’s participation in the paid workforce increased, their participation in household management activities has not decreased proportionally. In 1992, women spent more than double the amount of time (147 minutes/day) on domestic activities than men (37 minutes/day) (Australian Bureau of Statistics (ABS), 1998). In 2015-17 women were still spending almost double the amount of time in households where men were the main contributors to household income (131 minutes/day for men vs. 252 minutes/day for women), and approximately 30% more in households where women were the main contributors (163 minutes/day for men vs. 207 minutes/day for women), or both men and women contributed equally to household income (138 minutes/day for men vs. 198 minutes/day for women) (Wilkins et al., 2019).

Services and technology have also changed. In the 1990s, many supermarkets had restrictions on their opening hours, but by 2020, supermarkets were open seven days a week and many offered extended shopping hours. While in the 1990s there were still some instances of milkmen and green grocers’ delivering food to the door, this had been replaced with home delivery of ingredients, and takeaway meals in 2020. In the 1990s, ready-meals or partially prepared meals existed, but not to the same extent of meal box schemes in the 21^st^ century. While personal computers and the internet were starting to be introduced into family households in the 1990s, in 2020 it was not uncommon for personal computers, or other electronic devices such as smartphones or tablets, to be available to each member of the family (Australian Bureau of Statistics (ABS), 2018). By 2020 the internet boasted a plethora of websites and applications for assisting with planning, purchasing and preparation of meals (Doub et al., 2016). Additionally, the creation of electronic voice activated Artificial Intelligence (AI) devices, such as Amazon’s ‘Alexa’, or Google’s ‘Google Assistant’, increased the accessibility of these services for contemporary families.

Additionally, it should be noted that the cost of living has increased in Australia over this thirty-year period. Australian household expenditure on goods and services increased by 137% from 1993-1994 to 2015-2016 (Australian Bureau of Statistics (ABS), 2017b). The amount of money Australians were spending on food on average rose from AU$111 per week in 1993-1994 to AU$237 in 2015-2016. However, the proportion of total household expenditure spent on food decreased from 19% in the 1990s to 16.6% in 2015-2016 (Australian Bureau of Statistics (ABS), 2017b). Rates of eating outside of the home increased over this time period, and the per capita expenditure on meals eaten in cafes and restaurants increased by 30% from 1985 to 2006 (Australian Bureau of Statistics (ABS), 2007). Between 2005 and 2016, the average household spent an average of AU$12 more per week on meals eaten in cafes and restaurants (Australian Bureau of Statistics (ABS), 2017b), indicating that both the frequency and the cost of eating out has continued to rise over this time.

This information aims to provide context to the comparison of changes to the family meal presented in the manuscript, and to aid in situating and interpreting the findings across time.

References

Australian Bureau of Statistics (ABS). (1994). *Household and family trends in Australia*. ABS. Retrieved 4th March from <https://www.abs.gov.au/ausstats/abs@.nsf/featurearticlesbytitle/72DC873D21F1E2ECCA2569DE00221C82?OpenDocument>

Australian Bureau of Statistics (ABS). (1995). *Living arrangements: Children in families*. ABS. Retrieved 4th March from <https://www.abs.gov.au/ausstats/ABS@.nsf/2f762f95845417aeca25706c00834efa/a704eb29681a15ecca2570ec007517fc!OpenDocument>

Australian Bureau of Statistics (ABS). (1998). *How Australians use their time, 1997*. ABS. Retrieved 4th March from <https://www.abs.gov.au/AUSSTATS/abs@.nsf/Lookup/4153.0Main+Features11997?OpenDocument>

Australian Bureau of Statistics (ABS). (2007). *Trends in household consumption*. ABS. Retrieved 8th June from <https://www.abs.gov.au/ausstats/abs@.nsf/latestproducts/0485BB5550FE5799CA25732C00207C77?opendocument#Data%20sources%20and%20definitions>

Australian Bureau of Statistics (ABS). (2010). *Population by age and sex, Australian States and Territories, Jun 2010*. ABS. Retrieved 1st March from <https://www.abs.gov.au/ausstats/abs@.nsf/mf/3201.0>

Australian Bureau of Statistics (ABS). (2016). *2016 Census QuickStats: South Australia*. ABS. Retrieved 1st March from <https://quickstats.censusdata.abs.gov.au/census_services/getproduct/census/2016/quickstat/4>

Australian Bureau of Statistics (ABS). (2017a, 12 July 2019). *2016 Census QuickStats: Australia*. ABS. Retrieved 25th September from <https://quickstats.censusdata.abs.gov.au/census_services/getproduct/census/2016/quickstat/036>

Australian Bureau of Statistics (ABS). (2017b). *Household expenditure survey, Australia: Summary of results*. ABS. Retrieved 8th June from <https://www.abs.gov.au/statistics/economy/finance/household-expenditure-survey-australia-summary-results/latest-release#average-household-spending>

Australian Bureau of Statistics (ABS). (2018). *Household use of information technology*. ABS. Retrieved 9th August from <https://www.abs.gov.au/statistics/industry/technology-and-innovation/household-use-information-technology/latest-release>

Australian Bureau of Statistics (ABS). (2019). *Regional population by age and sex*. ABS. Retrieved 8th December from <https://www.abs.gov.au/statistics/people/population/regional-population-age-and-sex/latest-release#key-statistics>

Australian Bureau of Statistics (ABS). (2020). *Labour force status of families*. ABS. Retrieved 29th October from <https://www.abs.gov.au/statistics/labour/employment-and-unemployment/labour-force-status-families/latest-release>

Castles, I. (1993). *Census characteristics of South Australia* (1991 Census of Population and Housing, Issue.

Doub, A. E., Small, M. L., Levin, A., LeVangie, K., & Brick, T. R. (2016). Identifying users of traditional and Internet-based resources for meal ideas: An association rule learning approach. *Appetite*, *103*, 128-136. <https://doi.org/10.1016/j.appet.2016.04.006>

Greenville, J., Pobke, C., & Rogers, N. (2013). *Trends in the Distribution of Income in Australia*. <https://www.pc.gov.au/research/supporting/income-distribution-trends/income-distribution-trends.pdf>

Warren, D., Qu, L., & Baxter, J. (2020). *How we worked* (Australian Families Then & Now, Issue.

Wilkins, R., Laß, I., Butterworth, P., & Vera-Toscano, E. (2019). *The Household, Income and Labour Dynamics, in Australia survey: Selected findings from waves 1 to 17* (The 14th Annual Statistical Report of the HILDA Survey, Issue.
