# Supplementary material for: The family meal, a ritual frozen in time; an Australian grounded theory study
Source: Health Promot Int. 2023 Oct 4;38(5):daad124. doi: 10.1093/heapro/daad124 (PMC10548410; doi:10.1093/heapro/daad124)
Supplement: daad124_suppl_Supplementary_Files_2 [file daad124_suppl_supplementary_files_2.docx]

# Supplementary File 2

Demographic characteristics of family meal interview participants in 1990s and 2020

All data presented as n/total, unless otherwise specified

| **Participant characteristics** | | |
| --- | --- | --- |
|  | **1990s TOTAL participants n=32** | **2020 TOTAL participants n=22** |
| **Gender of adults** |  |  |
| - Male | 16/32 | 10/22 |
| - Female | 16/32 | 12/22 |
| **Age of adults (years)** mean (range) | 38 (26-46) | 43 (34-55) |
| **Highest level of education*** |  |  |
| - Secondary school | 13/32 | 2/22 |
| - Some tertiary education | 0 | 3/22 |
| - Trade or business qualification | 4/32 | 0 |
| - Degree or tertiary diploma | 10/32 | 16/22 |
| - Higher Degree | 4/32 | 1/22 |
| **Employment status** |  |  |
| - **Paid employment** | **20/32** | **19/22** |
| - - Females | 8/16 | 9/12 |
| - - Males | 12/16 | 10/10 |
| - **Homemaker** | **7/32** | **2/22** |
| - - Females | 7/16 | 2/12 |
| - - Males | 0 | 0 |
| - **Unemployed** | **5/32** | **1/22** |
| - - Females | 1/16 | 1/12 |
| - - Males | 4/16 | 0 |
| **Family characteristics** | | |
|  | **1990s TOTAL families n=16** | **2020 TOTAL families n=12** |
| - Two-parent family - Single-parent family | 15/16  1/16 | 10/12  2/12 |
| **Number of children living at home** mean (range) | 2.4 (1-3) | 2.4 (1-4) |
| **Age in years of children living at home** number (% of sample)   - 0-2 years - 3-5 years - 6-10 years - 11-15 years - 16-18 years - >18 years | 2 (5%)  9 (24%)  16 (42%)  7 (18%)  2 (5%)  2 (5%) | 1 (3%)  4 (14%)  10 (34%)  9 (31%)  2 (7%)  3 (10%) |
| **Household employment status** |  |  |
| - Two parents employed | 7/16 | 8/12 |
| - One parent employed | 6/16 | 3/12 |
| - Neither parent employed | 3/16 | 1/12 |
| **Household status*** |  |  |
| - Provided by state | 0 | 1/12 |
| - Renting from housing trust | 4/16 | 1/12 |
| - Renting privately | 3/16 | 2/12 |
| - Paying off mortgage | 3/16 | 5/12 |
| - Outright owners | 5/16 | 3/12 |
| **Annual household income*^a^** |  |  |
| - Lowest quintile | 0 | 0 |
| - Second quintile | 2/16 | 2/12 |
| - Third quintile | 3/16 | 1/12 |
| - Fourth quintile | 5/16 | 5/12 |
| - Highest quintile | 3/16 | 3/12 |

*Missing data for level of education n=1, household status n=1, household income n=4

^a^ Quintile’s based on census household forms in 1991 and 2016
